# Abdominal Aorta Balloon Occlusion Versus Standard Care in Placenta Accreta Spectrum Disorder: A Randomized Controlled Trial

**DOI:** 10.3390/life16071106

**Published:** 2026-07-02

**Authors:** Gauri Bapayeva, Meruyert Abdukassimova, Nazira Kadroldinova, Kuat Kassymbek, Karlygash Togyzbayeva, Lyazzat Saidildina, Viktor Zemlyanskiy, Kuanysh Balgynbayev, Mariya Usseyeva, Gulzhanat Aimagambetova, Milan Terzic

**Affiliations:** 1 Clinical Academic Department of Women’s Health, Corporate Fund University Medical Center, Astana Z05G9F0, Kazakhstan; gauri.bapaeva@umc.org.kz (G.B.); kuat.kassymbek@alumni.nu.edu.kz (K.K.); karligash.togyzbaeva@umc.org.kz (K.T.); lyazzat.saidildina@umc.org.kz (L.S.); gulzhanat.aimagambetova@nu.edu.kz (G.A.); milan.terzic@nu.edu.kz (M.T.); 2 Department of Surgery, School of Medicine, Nazarbayev University, Astana Z05P3Y4, Kazakhstan; nazira.kadroldinova@nu.edu.kz; 3 Clinical Academic Department of Radiology and Nuclear Medicine, Corporate Fund University Medical Center, Astana Z05G9F0, Kazakhstan; zemlyanskiy.viktor@umc.org.kz; 4 Clinical Academic Department of Anaesthesiology and Intensive Care, Corporate Fund University Medical Center, Astana Z05G9F0, Kazakhstan; k.balgynbaev@umc.org.kz; 5 Clinical Academic Department of Pathology, Corporate Fund University Medical Center, Astana Z05G9F0, Kazakhstan; mariya.useeva@umc.org.kz

**Keywords:** placenta accreta spectrum, abdominal aortic balloon occlusion, cesarean delivery, obstetric hemorrhage, hysterectomy

## Abstract

Placenta accreta spectrum (PAS) is associated with substantial intraoperative hemorrhage. Abdominal aortic balloon occlusion (AABO) has been introduced to mitigate blood loss, though its benefit remains debated. This study, registered at ClinicalTrials.gov (NCT06721182), assessed maternal outcomes in women with PAS undergoing cesarean delivery managed either with abdominal aortic balloon occlusion or standard surgical care alone. The study (*n* = 65) and control (*n* = 75) groups were randomly assigned. Primary outcomes were estimated intraoperative blood loss and transfusion requirements. Secondary outcomes included operative time, hysterectomy, intensive care unit (ICU) admission, length of hospitalization, and complications. Management with AABO was associated with a reduction in estimated intraoperative blood loss (1433.08 ± 1064.97 mL for AABO vs. 2213.33 ± 1574.06 mL in control) and lower transfusion requirements (687.48 ± 979.23 mL for AABO vs. 1161.84 ± 1140.33 mL in control). The incidence of hysterectomy was lower among patients managed with AABO; while this difference was not statistically significant, it was regarded as clinically important. No significant differences were observed in ICU admission rates, duration of hospital stay, or operative time. Procedure-related complications were observed. AABO was associated with lower perioperative blood loss and transfusion requirements, without an increase in overall complications.

## 1. Introduction

Obstetric hemorrhage is one of the leading causes of maternal mortality and morbidity worldwide. One of the risk factors of obstetric hemorrhage includes placental pathologies such as placenta previa and placenta accreta spectrum (PAS) disorders, the frequency of which is increasing throughout the world and accounts for one in 200 pregnancies for placenta previa, and it is difficult to estimate due to variable population and diagnostic criteria used for placenta accreta spectrum disorder [1,2,3]. PAS is accompanied by intraoperative massive bleeding with hemorrhagic shock and removal of the reproductive organ during cesarean section [4]. The most common risk factor for PAS is a uterine scar after a cesarean section. The risk of PAS increases with the increased number of prior cesarean sections and the presence of placenta previa or low-lying placenta [5]. Given the increasing frequency of cesarean sections worldwide [6], an increase in the incidence of PAS and associated complications can be expected.

Preoperative vascular control, such as uterine artery embolization or balloon occlusion, is increasingly employed to minimize hemorrhage. International clinical guidelines highlight the role of interventional modalities as an adjunctive management of PAS disorders [7,8,9]. Endovascular management of PAS includes prophylactic and resuscitative balloon occlusion with interventional radiological techniques or ultrasound-guided balloon placement and primary uterine artery embolization. The balloon placement for PAS may involve different arteries, including internal iliac arteries (IIABO), common iliac arteries (CIABO), abdominal aorta (AABO), and uterine arteries (UABO), with or without embolization [10]. Among these intravascular interventions, the use of aortic balloon occlusion is among the promising techniques [8,9,11,12,13,14]. Abdominal aorta balloon occlusion (AABO) is a minimally invasive procedure in which a balloon catheter is inserted into the infrarenal aorta or Zone III to control bleeding [15]. The use of this procedure does not require angiographic equipment or radiation exposure and can be performed in a standard operating theatre with no transportation between procedures [13,16,17]. The international guidelines recommend multidisciplinary team (MDT) decisions for the use of AABO for PAS for high-risk maternal hemorrhage cases [17].

Previous studies on the effectiveness of this endovascular intervention were mostly retrospective and lacked sufficient data from randomized clinical trials (RCTs). In the current study, we conducted an RCT in the tertiary care center with specialization in diagnosing and managing abnormal placentation. Patients with PAS are referred to the study center from all regions of the Republic of Kazakhstan. All PAS cases are managed by MDT, which includes a senior obstetrician team who perform all PAS cesarean sections, senior anesthesiologists, interventional radiologists, an obstetric ultrasound expert, a gynecologist, and a neonatologist.

This research project aims to assess the effectiveness of aortic balloon occlusion in minimizing blood loss and preserving the uterus during cesarean delivery with AABO. The central hypothesis proposes that aortic balloon occlusion can significantly reduce intraoperative bleeding during cesarean sections, lower the requirement for blood transfusions, decrease the incidence of hysterectomy, and shorten both the duration of surgery and the overall hospital stay among patients diagnosed with PAS disorders.

## 2. Materials and Methods

This prospective RCT studied the effect of aortic balloon occlusion of the aorta for delivery in cases of placenta accreta spectrum disorders in comparison to standard management. This study was registered in the ClinicalTrials.gov database (registration ID NCT06721182), and the study protocol was published previously [11]. It took place at Mother and Child Center, University Medical Center (UMC), Astana, Kazakhstan. The overall percentage of cesarean sections in our tertiary care center is up to 38% compared to 23% at the national level.

Study participants were pregnant patients referred to UMC for delivery with an antenatal PAS diagnosis. Diagnosis of PAS with complete previa was confirmed by a senior sonologist with over 10 years of experience with Philips ultrasound system (Philips Healthcare, Best, The Netherlands) equipped with a C9-4v endovaginal transducer (9–4 MHz) and a C5-1 convex abdominal transducer (1–5 MHz) on the basis of sonographic markers such as placental lacunae, disappearance of the normal hypoechoic retroplacental zone, abnormal structure of the border between the uterus and the bladder wall, and a pathological pattern of blood flow with color Doppler [11]. Placental edges were mapped preoperatively for each patient as well. At hospitalization, patients were screened against eligibility and exclusion criteria, outlined in the published study protocol [11]. Eligible participants were women aged 18–45 years with a singleton pregnancy ≥ 34 weeks’ gestation, a confirmed diagnosis of any grade placenta accreta spectrum and complete previa, an indication for elective cesarean section, and consent for aortic balloon occlusion. Exclusion criteria included gestational age < 34 weeks, multiple pregnancies, emergency cesarean section, coagulation disorders (including abnormal preoperative activated partial thromboplastin time (aPTT), prothrombin time (PT), or international normalized ratio (INR)), use of antiplatelet or anticoagulant therapy, and a history of thrombotic events. Recruitment was conducted between August 2024 and January 2026.

The sample size determination was done a priori, taking into account the estimated differences in the volume of blood loss, blood transfusion, and hysterectomy rate from earlier research. The sample size of 144 individuals (72 per group) was expected to provide 80% power to identify any difference at the 0.05 level of significance. This statistical framework corresponds to our previously published study protocol [11].

Enrolled patients were randomized consecutively using simple randomization with an intended 1:1 allocation ratio. Although randomization was performed on a one-to-one basis, the final group sizes differed due to post-randomization exclusions occurring exclusively in the intervention arm. These exclusions were related primarily to withdrawal of consent for balloon occlusion and clinical considerations prioritizing maternal safety that precluded application of the intervention. Participants allocated to the standard caesarean section proceeded as randomized, resulting in a larger control group. Blinding of the surgical team was not feasible due to the nature of the intervention.

As part of preoperative planning, patients allocated to the AABO group underwent magnetic resonance imaging (MRI) of the abdomen and pelvic organs using a Philips Ingenia 3.0 T system (Philips Healthcare, Best, The Netherlands). These images were used to calculate the aortic diameter at the intended site of balloon inflation and to measure the length of the iliac artery from the anticipated puncture site to the infrarenal aortic occlusion zone. This morphometric assessment allowed for the precise calculation of the required insertion depth and the optimal balloon diameter (Mammoth™ PTA 0.035-inch 16–30 × 40 mm balloon catheter; Meril Life Sciences Pvt. Ltd., Vapi, Gujarat, India) to ensure safe and stable placement. MRI data were not used for the assessment of placental invasion and for surgical planning.

Balloon placement was performed by an interventional radiologist immediately prior to commencement of cesarean section in the operating room under ultrasound (US) guidance and under local anesthesia of a lidocaine 1–10 mL solution. Access to Zone IIIa of the abdominal aorta was reached through the right common femoral artery. The catheter was flushed with heparinized saline, and balloon was inflated immediately to the precalculated diameter (according to aorta MRI) after the obstetricians had delivered the newborn for a maximum of 25 min. If hemostasis was not reached in that timeframe, the balloon was re-inflated after a break of two minutes. Deflation was performed gradually under the control of blood pressure. Manual compression of the puncture site for 10 min was performed after removal of the sheath.

Patients in the control group underwent standard management according to the national guideline without placement of an aortic balloon using a one-step conservative approach, which involves uterine incision above the placenta and placental removal under direct visualization with en bloc resection of the affected uterine wall, followed by uterine reconstruction (metroplasty) in cases where placental separation is not feasible or hysterectomy when the affected area was excessive. The same experienced surgical team performed the procedures in both groups to minimize operator variability. Additional hemostatic procedures were applied to both groups as required, including supplemental hemostatic sutures, B-lynch and O’Leary sutures, and pharmacological agents (oxytocin, methylergometrin, carbetocin (Pabal^®^; Ferring Pharmaceuticals, Saint-Prex, Switzerland), and tranexamic acid). Standard intraoperative hemorrhage prophylaxis included intravenous oxytocin 10 international units (IU) bolus after fetal delivery, followed by slow intravenous infusion of 20 IU of oxytocin during the remainder of the operation. Additional hemostatic agents, including methylergometrine, carbetocin, and tranexamic acid, were administered at the surgeon’s discretion. The thromboprophylaxis regimen after surgery was enoxaparin 40 mg given 12 h after surgery and continued for at least 5 days or until discharge from the hospital in both groups.

Adverse events were defined as any intraoperative or postoperative complication occurring during hospitalization. These were intraoperative surgical complications (bladder and bowel injury), postoperative complications (hematometra, infection, acute kidney injury, intestinal paresis, and reintervention), and procedure-specific complications from arterial catheterization (thrombosis of arteries and veins, hematoma, and phlebitis).

Adverse events were systematically evaluated for all participants by reviewing operative notes, anesthesia records, postoperative clinical records, and inpatient medical records. Complications were grouped as intraoperative complications, postoperative complications, and catheter-related complications for analysis. To prevent double-counting of complications, analysis of adverse events was done on a per-patient basis, where each patient contributed only once per category of complications regardless of the number of complications.

Statistical analyses compared women managed with aortic balloon occlusion (case) with those receiving standard care (control). All analyses were conducted according to the per-protocol principle. Continuous and ordinal variables were summarized as mean ± standard deviation (SD) and median with interquartile range (Q1–Q3). Categorical variables were described as counts and percentages. All tests were two-sided. The primary outcome was intraoperative blood loss (mL), whereas secondary outcomes included perioperative total transfusion volume, hysterectomy, duration of surgery, intensive care unit (ICU) and overall hospital length of stay, neonatal Apgar scores at one and five minutes, neonatal intensive care unit (NICU) days, and perioperative complications. The estimated blood loss during surgery was calculated quantitatively based on the weight difference in the dry versus blood-soaked surgical sponges used, as well as the quantity of blood obtained through cell salvaging. The severity of PAS according to the International Federation of Gynecology and Obstetrics (FIGO) classification [3] was assessed based on histopathological analysis when implantation site tissues were available (hysterectomy or partial uterine wall resection), and clinical assessment during surgery if histopathological evaluation was not possible. Assessment of PAS severity intraoperatively was done by the operating surgeon using the degree of placental invasion, as well as infiltration of the adjacent structures [3].

For baseline and outcome comparisons involving continuous variables, distributional assumptions were evaluated. When both normality and homoscedasticity were reasonably satisfied, group differences were assessed with independent-samples *t*-tests; otherwise, the non-parametric Mann–Whitney U test was applied. Baseline categorical characteristics and categorical perioperative outcomes were compared using Pearson’s χ^2^ test or Fisher’s exact test when expected counts were small. Effect sizes were reported where appropriate to aid clinical interpretation of continuous perioperative outcomes (e.g., blood loss, surgery duration, transfusion volumes, ICU/NICU/hospital days, Apgar scores), and raw *p*-values from group comparisons were adjusted for multiple testing using the Benjamini–Hochberg false discovery rate (FDR-BH) procedure. The same procedure was applied to categorical perioperative outcomes. Two-sided *p*-values < 0.05 after FDR correction were considered statistically significant. To characterize interrelationships among treatment group, PAS severity, and key clinical outcomes, a mixed-type correlation matrix was constructed, with Spearman’s rank correlation coefficients for the pairs of continuous or ordinal variables, rank-biserial correlations for binary–continuous pairs, and φ for binary-binary variable pairs.

The effect of AABO on intraoperative blood loss and total transfusion volume was further evaluated using linear regression on log-transformed outcomes. Adjusted regression models were pre-specified to account for the imbalanced prognostic factors and to evaluate the robustness of the treatment effect. Three prespecified models were fitted for each endpoint (unadjusted; adjusted for PAS grade; and adjusted for PAS grade plus number of prior caesarean sections) using HC3 heteroskedasticity-robust standard errors. Exponentiated coefficients are reported as ratios of geometric means with 95% confidence intervals (CIs). The association between AABO and hysterectomy was analyzed using logistic regression (crude and adjusted for PAS grade and prior caesarean sections), with odds ratios (ORs) and 95% CI.

Statistical analysis was performed in Python 3.12.12 (Python Software Foundation, Wilmington, DE, USA). Data processing was conducted using pandas 2.2.2 and numpy 2.0.2; statistical analyses were conducted with SciPy 1.16.3 and statsmodels 0.14.5, and visualizations were created with matplotlib 3.10.0 and seaborn 0.13.2.

## 3. Results

### 3.1. Participant Flow

**Figure 1:**
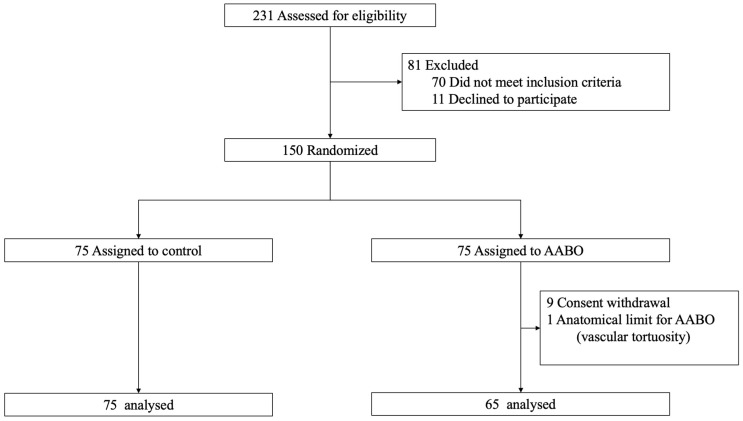
Consolidated standards of reporting trials (CONSORT) flow diagram. CONSORT flow diagram showing participant enrollment, randomization, post-randomization exclusions, and per-protocol analysis population. Post-randomization exclusions occurred only in the abdominal aorta balloon occlusion (AABO) arm (*n* = 10); reasons are shown.

A total of 231 patients referred to the study hospital with antenatal diagnoses of PAS with placenta previa during the study period from August 2024 to January 2026 were considered for eligibility, out of which 81 patients were excluded from the study due to not fulfilling the inclusion criteria or participation refusal. A total of 150 patients were randomly assigned to the intervention group, i.e., cesarean delivery with AABO, or the control group, i.e., cesarean section alone. Out of the 75 patients in the intervention group, nine patients refused to undergo AABO post-randomization and withdrew consent. One of the patients was identified to have tortuous blood vessels on MRI angiography that precluded safe advancement of the balloon catheter and, therefore, safe application of the intervention. Thus, a total of 65 patients in the intervention group received AABO. All patients in the control group were delivered via standard cesarean section, as explained in detail in Section 2. Participant losses and exclusion reasons are depicted in the Consolidated Standards of Reporting Trials (CONSORT) flow diagram (Figure 1).

### 3.2. Study Participants’ Baseline Characteristics

Baseline demographic and clinical characteristics of the analyzed population are presented in Table 1 and Table 2. Generally, the AABO and control groups were broadly comparable across most measured variables. Age, body mass index (BMI), gestational age at delivery, and neonatal birth weight were similar between groups, with small standardized mean differences (SMDs), indicating minimal imbalance. The distribution of placenta accreta spectrum (PAS) severity, as assessed by pathological and/or clinical grading, was also similar between groups (SMD = 0.065). Most categorical variables, including ethnicity, marital status, employment status, smoking, prior intrauterine interventions, and surgeon distribution, demonstrated small to moderate imbalances (generally SMD < 0.30), suggesting reasonable comparability across these characteristics.

Clinically meaningful imbalances were observed in several obstetric history characteristics. In particular, gravidity (SMD = 0.43), parity (SMD = 0.59), and number of prior cesarean sections (SMD = 0.51) were higher in the AABO group, indicating a greater burden of prior pregnancies and surgical history in this group. An additional moderate imbalance was noted for conception via assisted reproductive technologies (ART).

### 3.3. Intraoperative Blood Loss

Intraoperative blood loss was substantially lower in the AABO group than in the control group (see Table 3). The median blood loss was 1000 mL (Q1–Q3: 700–2000 mL) with balloon occlusion, whereas in the control group, women lost 1600 mL (Q1–Q3: 1000–3100 mL) of blood during surgery, corresponding to a Hodges–Lehmann estimated median difference of −500 mL (95% CI −800 to −200 mL; *p* _FDR-BH_ = 0.0066). In the mixed-effect correlation matrix, AABO showed a similar moderate negative association with intraoperative blood loss ($r_{rb}=-0.33$, *p* _FDR-BH_ =0.002), reinforcing the pattern of systematically lower bleeding among patients receiving AABO.

Due to the right-skewed distribution of intraoperative blood loss, analyses were conducted on the log scale to better meet model assumptions. Robust (HC3) standard errors were used to ensure valid inference under potential heteroskedasticity.

The PAS grade was included in adjusted models, as PAS was strongly associated with intraoperative bleeding (Spearman’s ρ=0.58, *p* _FDR-BH_ <0.001). Adjusting for PAS improved model precision, reduced unexplained heterogeneity, and allowed estimation of the AABO effect at comparable levels of placental invasion, which increases both statistical precision and clinical interpretability. Although groups were generally comparable, baseline imbalances were observed in gravidity, parity, and number of prior caesarean deliveries. Given the collinearity between these variables, the number of prior caesarean sections was selected as the most clinically relevant obstetric baseline factor for adjustment, primarily to improve precision and to evaluate the robustness of the treatment effect.

Model diagnostics did not indicate undue influence of individual observations. Influence measures, including Cook’s distance and DFBETAs, were within acceptable ranges, and no influential outliers or high-leverage points were identified.

Across all three specified models (unadjusted, PAS-adjusted, and PAS and prior caesarean-adjusted), AABO was associated with a 33–36% lower geometric mean intraoperative blood loss compared with the control group, and this estimate was consistent after adjustments for PAS grade and prior caesarean sections. In the primary adjusted model, the ratio of geometric mean blood loss between the AABO and control groups was 0.64 (95% CI 0.53–0.77; *p* < 0.001), corresponding to an approximate 36% lower intraoperative blood loss with AABO. The results were stable across prespecified models and were not influenced by outliers or leverage points (Supplementary Table S1).

### 3.4. Perioperative Total Transfusion Volume

The total transfusion volume was considerably lower in the AABO group than in the control group. The mean total perioperative transfusion volume was 687 ± 979 mL in the AABO group versus 1162 ± 1140 mL in controls, with medians of 0 mL (Q1–Q3: 0–1064 mL) and 707 mL (Q1–Q3: 0–2126.5 mL), respectively (see Table 3). The Hodges–Lehmann estimated median difference was −205 mL (95% CI −650 to 0 mL; *p* _FDR-BH_ = 0.0154). Moreover, according to the mixed-method correlation matrix, AABO demonstrated a moderate negative association with the total transfusion volume ($r_{rb}=-0.28$, *p* _FDR-BH_ =0.007).

The total transfusion volume also showed a moderate positive correlation with PAS grade (Spearman’s ρ=0.54, *p* _FDR-BH_ <0.001) and a weaker but statistically significant correlation with the number of prior caesarean sections (ρ=0.20, *p* _FDR-BH_ =0.025). These associations motivated adjustment for PAS grade and prior caesarean history in the multivariable models aimed at evaluating the effect of AABO on transfusion requirements.

In the primary model for log-transformed total transfusion volume (Supplementary Table S2), which included AABO status, PAS grade, and number of prior caesarean sections, AABO was significantly associated with reduced transfusion requirements. The coefficient for AABO on the log-scale was −1.81 (robust SE = 0.499, 0.0003; 95% CI −2.790 to −0.835), corresponding to a ratio of geometric means of 0.16 (95% CI 0.06–0.43). Thus, after adjustments to PAS grade and prior c-sections, patients managed with AABO required, on average, about 84% less volume of perioperative transfusions than the controls. The PAS grade showed a large positive association with transfusion (β = 1.51, robust SE 0.23, *p* < 0.001), and the number of prior caesarean sections had a smaller, borderline-significant effect (β = 0.40, p = 0.063), suggesting a possible but less certain contribution to transfusion needs during the present c-section surgery.

### 3.5. Association Between AABO and Hysterectomy

Hysterectomy occurred in 15/65 women (23.1%) in the AABO group and 22/75 (29.3%) in the control group (see Table 4). The estimated risk ratio was 0.79 (95% CI 0.45–1.39), corresponding to an absolute risk difference of −6.3 percentage points (95% CI −25.9% to 14.4%). However, a group-comparison χ^2^ test showed no evidence of association between AABO use and hysterectomy (χ^2^ = 0.70, *p* _FDR-BH_ = 0.67). Consistently, in a univariable logistic regression, AABO was not associated with hysterectomy (crude OR 0.72, 95% CI 0.34–1.55, *p* = 0.40), and this remained non-significant after adjustment for PAS grade and number of previous caesarean sections (adjusted OR 0.60, 95% CI 0.23–1.54, *p* = 0.28).

The frequency of metroplasty (see Table 4) was numerically higher in the AABO group (69.2%) than in the standard care group (53.3%), with a risk ratio of 1.30 (95% CI 0.99–1.69) and an absolute risk difference of 15.9 percentage points (95% CI −7.0% to 37.0%). However, this difference was not statistically significant (χ^2^ = 3.69, *p* _FDR-BH_ = 0.27).

### 3.6. Other Outcomes

**Figure 2:**
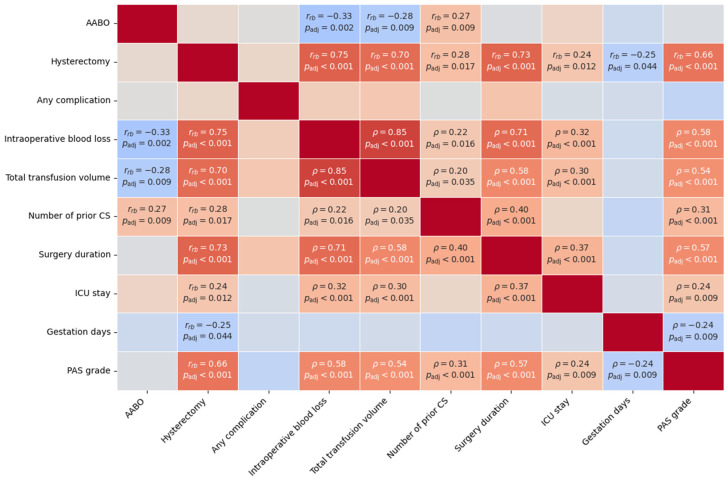
Pairwise correlations among key clinical variables. Effect-size measures: Spearman’s ρ for continuous–continuous pairs; rank-biserial correlation (r_rb_) for binary–continuous pairs; phi (φ) for binary–binary pairs. Only correlations with FDR-adjusted *p* < 0.05 are shown. The color gradient represents the direction and magnitude of the correlation coefficients, with warmer colors indicating positive correlations and cooler colors indicating negative correlations, and greater color intensity indicating stronger correlations. AABO, abdominal aortic balloon occlusion; PAS, placenta accreta spectrum; CS, cesarean section; ICU, intensive care unit.

Perinatal and neonatal outcomes were similar between the balloon occlusion (*n* = 65) and control (*n* = 75) groups after FDR-BH correction (see Table 3). The median surgery duration did not differ materially (73.0 [53.0–95.0] vs. 72.0 [59.0–89.0] minutes; *U* = 2386.5, *p* _FDR-BH_ = 0.98). Length of ICU and hospital stay outcomes were also comparable, with a median ICU stay of one day in both groups (*U* = 2749.5, *p* _FDR-BH_ = 0.24) and a median hospital stay of 7 days in each group (*U* = 2682.0, *p* _FDR-BH_ = 0.61). NICU stay among neonates admitted did not differ significantly across study groups (*U* = 2316.0, *p* _FDR-BH_ = 0.95) (see Table 3). Neonatal condition at birth was similar, with overlapping distributions of both 1 min Apgar scores (median of 7.0 [6.0–7.0] in both study arms, *U* = 2460.5, *p*_FDR-BH_ = 0.98) and 5 min Apgar scores (median of 7.0 [7.0–8.0] in both study arms, *U* = 2431.0, *p* _FDR-BH_ = 0.98). Pairwise correlations among key clinical variables are summarized in Figure 2.

### 3.7. Adverse Effects

Intraoperative complications, which included bladder and intestinal injury, occurred in 13 (20.0%) and 11 (14.7%) of women in the AABO and control groups, respectively (χ^2^ = 0.70; *p*_FDR-BH_ = 0.5046) (see Table 4). Postoperative complications included acute kidney injury secondary to hemorrhage and hypoperfusion (one case), hematometra (two cases), surgical site infection (one case), intestinal paresis (one case), arterial and venous thromboses (five cases), relaparotomy (two cases) due to postoperative hemorrhage with hysterectomy to control ongoing bleeding, and suction evacuation of intrauterine contents (one case). These occurred in 7 of 65 women (10.8%) in the AABO group and 6 of 75 women (8.0%) in the control group (χ^2^ = 0.07; *p*_(FDR-BH)_ = 0.79). Catheter-related complications in the AABO group included two cases of external iliac artery thrombosis, with one having simultaneous thrombosis of the common iliac artery, one case of right common femoral vein thrombophlebitis, and two cases of great saphenous vein thrombosis. No thrombotic events were recorded in the control group. No maternal or neonatal deaths occurred in either group.

## 4. Discussion

In this study, the role of AABO in controlling massive hemorrhage for PAS cases was investigated. Our research findings support the results of previous studies that showed the safety and efficacy of the placement of abdominal aortic balloon occlusion in the setting of planned cesarean sections [15]. Among the key findings, AABO was associated with significantly reduced blood loss, both in absolute terms and in relation to transfusion requirements. The results of our study support a meaningful reduction of approximately 33% blood loss associated with AABO after accounting for PAS severity while confirming that higher PAS grades independently predict greater hemorrhage. In addition, holding the PAS grade constant, AABO use was associated with 79% lower transfusion volume on average. These results support the hypothesis that AABO contributes to blood conservation during cesarean delivery in patients with PAS. Other perioperative outcomes, including surgery duration, ICU days, NICU days, hospital stay duration, and Apgar scores, did not differ significantly between groups after correction for multiple testing. Effect sizes for these comparisons were generally small, suggesting clinically negligible differences. These results indicate that while AABO does not appear to affect neonatal or broader recovery outcomes directly, it may provide meaningful intraoperative hemostatic benefits, reducing both blood loss and the need for transfusion. This underscores AABO’s potential role as a targeted intervention in managing high-risk cesarean deliveries with abnormal placentation.

This research is a randomized prospective cohort study conducted for the first time in Central Asia. While most previous investigations focused on retrospective analyses, in this study, the main outcomes were compared prospectively. All study subjects were recruited from a tertiary care center in Astana, Kazakhstan. Patients are referred to this center from all regions of the country, which contributes to a diverse study population. At the same time, participants were initially allocated to the case and control groups randomly, reducing the risk of selection bias at enrollment. Baseline patient characteristics were broadly similar across many key categorical variables; however, some differences were observed after post-randomization exclusions. Post-randomization exclusions in the AABO arm (n = 10, 13.3% of those randomized to intervention) may introduce selection bias; however, adjusted analyses accounting for baseline imbalances yielded results consistent with the primary analysis, and the observed treatment effect is robust to this exclusion. Therefore, although randomization supports baseline comparability at allocation, observed differences in outcomes should be interpreted with consideration of potential residual imbalances in the analyzed cohort.

Anatomically, the abdominal aorta is divided into three zones, and occlusion of these zones leads to blockage of various aortic divisions and organs. Zone II and Zone III are the most of interest for pelvic surgeries, including cesarean section. Zone II or middle abdominal aorta includes the arterial section from the celiac artery to the lowest renal artery, and Zone III involves the section from the lowest renal artery to the aortic bifurcation [18]. Blockage of Zone II can be complicated with ischemia–reperfusion injury (IRI) if not limited to an occlusion time of 15 min [18], while occlusion of the abdominal aorta at Zone III has been shown to be superior to embolization balloon occlusion of other arteries, and it is less complicated compared with Zone II occlusion [19,20,21]. Infrarenal occlusion of the aorta or Zone III gives more control over collaterals and contributes significantly to decreasing intraoperative blood loss [15,22,23]. Occlusion of the abdominal aorta is performed under the control of ultrasound and does not require radiation exposure, which makes this technique safe for the mother and fetus. Interventional procedures allow a better operative field view due to the pelvic blood supply block [22]. Potential complications of this blood flow obstruction are organ ischemia and arterial thrombosis [11,22]. To prevent such complications, in this research, the maximum inflation time of the balloon was 25 min, after which the balloon was deflated for two minutes, and if necessary, the procedure was repeated again [11]. Consistent with previous findings [22], the current research found no evidence of either organ ischemia or reperfusion-related complications.

The effectiveness of abdominal aortic balloon occlusion is still controversial based on some existing research results [24,25,26]. However, most existing studies report a significant reduction in blood loss and red blood cell transfusion volume in patients with AABO. According to retrospective cohort evaluations, including 364 singleton pregnancies resulted in advantages of abdominal aortic occlusion in all clinical outcomes, including blood loss (1370.5 [752.0] ml vs. 3536.8 [1383.2] ml; *p* < 0.001), packed red blood cell transfusion (3.0 [4.0] vs. 13.8 [6.9], *p* < 0.001, respectively), and 95% of uterus preservation in the case group in comparison with 26.7% in the control group. In addition, operation times and ICU days were lower in the balloon group [27]. However, a study by Ye and colleagues involved balloon placement under fluoroscopic guidance [27]. In our investigation, we used no radiation exposure to our patients, and two groups were prospectively randomized. A study by Lu et al. [28] among the Chinese population evaluated the effectiveness of abdominal aortic placement and showed the effectiveness of the procedure, similar to our results. Similar findings are demonstrated in recent retrospective cohort studies carried out by Ioscovich et al. [16] among the Israeli population and Yin et al. [29], Zhao et al. [22], and Zheng et al. [30] among the Chinese population. As the first study of its kind in a Central Asian cohort, our prospective work demonstrates results that are broadly consistent with those reported in other countries, although most available studies are retrospective in nature. Although intraoperative blood loss and transfusion volume decreased significantly, there was no difference in hysterectomy rates between the two groups. In contrast, a study by Yang et al. [24], Li et al. [31], and Xie et al. [32] showed that AABO decreases hysterectomy rates, while there is no effect on blood loss and transfusion volume, suggesting that AABO is a safe interventional modality. Interestingly, a study by Huo et al. [26] showed increased blood loss and transfusion rate in the AABO group. Some of the inconsistencies seen between the results of our research and the results from previous studies might be due to the variations in the methodologies that were used. Our research utilized a randomized control trial, which provides prospective data on the impact of AABO in PAS treatment.

The present study did not identify a significant difference between the intervention and control groups in secondary outcomes (surgery duration, ICU stay, length of hospitalization, NICU stay, and Apgar scores). These data are comparable to previous research outcomes [27,28]. However, several studies have reported twice as few ICU admissions for the AABO group [33,34]. Our hospital admitted all patients to the ICU to ensure optimal monitoring and safety, with no difference in overall length of stay due to the presence of complications of different natures in both groups, which required prolonged ICU stay and hospitalization in both groups.

The nature of complications was expected, with few patients in the AABO investigation arm developing arterial and venous thrombosis, managed conservatively. Even though studies have reported safe continuous AABO up to 80 min, only one case of the longest AABO for 25 min in our investigation has resulted in thrombosis of the external iliac artery and common iliac artery. In the control group, two patients required relaparotomy with hysterectomy due to early postsurgical hemorrhage, and one developed acute kidney injury. Data for bladder injury rate were similar in both groups (10 AABO vs. eight controls). Some complications reported by other studies, such as intestinal obstruction and neurological complications of the lower limbs, were not encountered in the present study [16,18,28,35,36]. Our findings provide no evidence of increased peri- or postoperative morbidity associated with AABO; a shift from surgery-related complications towards procedure-related complications is seen.

AABO effectiveness was compared to the traditional surgical method in cases of PAS in our prospective randomized trial. To our knowledge, this is the first prospective randomized trial to evaluate this intervention. The prospective nature of this study, clear definition of inclusion and exclusion criteria, and predefined study outcomes facilitated non-biased analyses of data. Another strength of this study is our methodology’s reliability due to the same experienced team performing surgeries for cases and controls. This allows avoiding any intraoperative differences and reducing the risk of any biases connected with the skills of individual surgeons. Furthermore, as the placement of balloons was done under US guidance, this has removed the risk of radiation exposure to fetuses. Also, as US equipment is transportable to the operating room, there is no need to transfer patients from interventional radiology to the operating room, which allows for better maternal outcomes without delay.

Present research has several limitations, one of them being the presence of statistically significant differences between groups in baseline characteristics—higher numbers for gravidity, parity, and prior caesarean sections in the AABO group. Although patients were randomized, the AABO group tended to have slightly more complex obstetric histories, which places them at a higher risk of massive hemorrhage. Therefore, even though women in the AABO group were already at a disadvantage from the start, their reduced blood loss still indicates the efficiency of the technique. Another limitation of this study is the resulting wide variance in intraoperative blood loss and blood transfusion amount, which could be the result of the tremendous heterogeneity that exists within PAS surgeries. While a logarithmic transformation was performed in an attempt to lessen skewness, there were still large amounts of dispersion present. A further limitation is that 10 subjects were excluded following randomization. While these exclusions have been carried out according to set standards, post-randomization exclusions do have the potential for the introduction of bias. The limit of the single-center design is reduced generalizability; a multi-center design would allow better applicability of investigation data, as well as an increase in the number of participants. Due to the nature of the intervention, blinding of the surgical team was not possible, which might have led to bias.

## 5. Conclusions

This research has provided support for the use of balloon occlusion of the aorta for the management of PAS by reducing blood loss and reducing the requirement for red blood cell transfusion, yet the presence of complications highlights the importance of choosing candidates for the intervention who would benefit the most. Additional research is required to outline the characteristics of patients with the most beneficial outcome. Abdominal aortic balloon occlusion should be considered as part of an individualized surgical strategy, applied within experienced multidisciplinary teams rather than as a routine intervention. AABO is a promising management technique for cases of placenta accreta syndrome, which are predicted to rise in the future.

## Figures and Tables

**Table 1:** Baseline maternal and obstetric characteristics (numerical variables) by treatment group.

Variable	AABO (*n* = 65)	Control Group (*n* = 75)	Standardized Mean Difference
Age (years)			
Mean ± SD	34.53 ± 4.59	35.12 ± 5.79	−0.112
Median [Q1–Q3]	34.16 [32.00–37.60]	35.21 [32.21–38.82]	
BMI (kg/m^2^)			
Mean ± SD	30.91 ± 6.09	31.28 ± 6.55	0.058
Median [Q1–Q3]	30.00 [26.22–34.52]	29.67 [26.59–34.41]	
Gravidity			
Mean ± SD	5.23 ± 1.85	4.43 ± 1.90	0.429
Median [Q1–Q3]	5.00 [4.00–6.00]	4.00 [3.00–6.00]	
Parity			
Mean ± SD	2.94 ± 0.97	2.29 ± 1.21	0.586
Median [Q1–Q3]	3.00 [3.00–3.00]	2.00 [1.50–3.00]	
Number of prior c-sections			
Mean ± SD	2.23 ± 1.20	1.64 ± 1.13	0.508
Median [Q1–Q3]	2.00 [1.00–3.00]	1.00 [1.00–2.50]	
Gestation (days)			
Mean ± SD	253.74 ± 8.21	255.24 ± 7.71	−0.189
Median [Q1–Q3]	255.00 [249.00–259.00]	257.00 [251.00–260.00]	
Birth weight (g)			
Mean ± SD	2938.55 ± 491.69	3052.69 ± 461.93	−0.240
Median [Q1–Q3]	2940 [2700–3220.00]	2800.00–3350.00]	

Data are presented as mean ± standard deviation (SD) and median (Q1–Q3). Standardized mean differences (SMDs) are presented as measures of between-group balance; values greater than 0.10 are interpreted as indicating potential imbalance. AABO, abdominal aorta balloon occlusion; BMI, body mass index.

**Table 2:** Baseline maternal and obstetric characteristics (categorical variables) by treatment group.

Variable	Category	AABO (*n* = 65)	Control Group (*n* = 75)	Standardized Mean Difference
PAS grade	Accreta	38 (58.5%)	42 (56.0%)	0.065
	Increta	18 (27.7%)	21 (28.0%)	
	Percreta	9 (13.8%)	12 (16.0%)	
Mother ethnicity	Kazakh	59 (90.8%)	63 (84.0%)	0.204
	Other ethnicities	6 (9.2%)	12 (16.0%)	
Marital status	Single	2 (3.1%)	2 (2.7%)	0.233
	Married	63 (96.9%)	71 (94.7%)	
	Divorced	0 (0.0%)	2 (2.7%)	
Employment status	Unemployed	26 (40.0%)	24 (32.0%)	0.167
	Employed	39 (60.0%)	51 (68.0%)	
Smoking	No	64 (98.5%)	74 (98.7%)	−0.017
	Yes	1 (1.5%)	1 (1.3%)	
Prior intrauterine interventions *	No	38 (58.5%)	51 (68.0%)	−0.198
	Yes	27 (41.5%)	24 (32.0%)	
Conception type	Natural	64 (98.5%)	69 (92.0%)	0.303
	ART/IVF	1 (1.5%)	6 (8.0%)	
Intraoperative peritoneal adhesions	No	39 (60.0%)	36 (48.0%)	0.241
	Yes	26 (40.0%)	39 (52.0%)	

Data are presented as *n* (%). Standardized mean differences (SMDs) are presented as measures of between-group balance; values greater than 0.10 are interpreted as indicating potential imbalance. AABO, abdominal aorta balloon occlusion; PAS, placenta accreta spectrum; ART, artificial reproductive technologies; IVF, in vitro fertilization. * Prior intrauterine interventions refer to previous procedures involving instrumentation or surgical entry into the uterine cavity, including dilation and curettage, hysteroresectoscopy procedures (polypectomy and myomectomy), and myomectomy involving entry into the uterine cavity.

**Table 3:** Study outcomes across study groups (numerical).

Variable	Statistics	AABO	Control Group	Estimated Median Difference	*p* Value
		(*n* = 65)	(*n* = 75)		
Surgery duration (min)	Mean ± SD	76.05 ± 27.19	79.09 ± 31.93	−1.00 [−10.00–8.00]	*U* = 2386.500; *p*_FDR-BH_ = 0.9785
	Median [Q1–Q3]	73.00 [53.00–95.00]	72.00 [59.00–89.00]		
Intraoperative blood loss (mL)	Mean ± SD	1433.08 ± 1064.97	2213.33 ± 1574.06	−500.00 [−800.00–−200.00]	*U* = 1637.000; *p*_FDR-BH_ = 0.0066
	Median [Q1–Q3]	1000.00 [700.00–2000.00]	1600.00 [1000.00–3100.00]		
Total transfusion volume (mL)	Mean ± SD	687.48 ± 979.23	1161.84 ± 1140.33	−205.00 [−650.00–0.00]	*U* = 1764.500; *p* _FDR-BH_ = 0.0154
	Median [Q1–Q3]	0.00 [0.00–1064.00]	707.00 [0.00–2126.50]		
ICU stay (days)	Mean ± SD	1.49 ± 1.11	1.35 ± 1.74	0.00 [0.00–0.00]	*U =* 2749.500; *p*_FDR-BH_ = 0.2389
	Median [Q1–Q3]	1.00 [1.00–1.00]	1.00 [1.00–1.00]		
Hospitalization (days)	Mean ± SD	8.23 ± 3.30	7.69 ± 2.91	0.00 [0.00–0.00]	*U* = 2682.000; *p*_FDR-BH_ = 0.6068
	Median [Q1–Q3]	7.00 [6.00–9.00]	7.00 [5.50–9.00]		
NICU stay (days)	Mean ± SD	2.02 ± 2.20	2.39 ± 2.80	0.00 [0.00–1.00]	*U* = 2316.000; *p*_FDR-BH_ = 0.9537
	Median [Q1–Q3]	2.00 [0.00–2.00]	2.00 [0.00–3.00]		
1 min Apgar score	Mean ± SD	6.45 ± 1.00	6.40 ± 1.14	0.00 [0.00–0.00]	*U* = 2460.500; *p*_FDR-BH_ = 0.9785
	Median [Q1–Q3]	7.00 [6.00–7.00]	7.00 [6.00–7.00]		
5 min Apgar score	Mean ± SD	7.25 ± 0.87	7.29 ± 1.04	0.00 [0.00–0.00]	*U* = 2431.000; *p*_FDR-BH_ = 0.9785
	Median [Q1–Q3]	7.00 [7.00–8.00]	7.00 [7.00–8.00]		

Data are presented as mean ± standard deviation (SD) and median (Q1–Q3). Median differences were estimated using the Hodges–Lehmann method, with *p* values from the Mann–Whitney U test. AABO, abdominal aorta balloon occlusion; ICU, intensive care unit; NICU, neonatal intensive care unit.

**Table 4:** Study outcomes across study groups (categorical).

Variable	Category	AABO (*n* = 65)	Control Group (*n* = 75)	Effect Estimate (95% CI)	*p* _FDR-BH_
Hysterectomy	No	50 (76.9%)	53 (70.7%)	RR: 0.79 (0.45–1.39) RD: −6.3% (−25.9–14.4%)	0.6707
	Yes	15 (23.1%)	22 (29.3%)		
Metroplasty	No	20 (30.8%)	35 (46.7%)	RR: 1.30 (0.99–1.69) RD: 15.9% (−7.0% to 37.0%)	0.2738
	Yes	45 (69.2%)	40 (53.3%)		
Intraoperative complications ^1^	No	52 (80.0%)	64 (85.3%)	RR: 1.36 (0.66–2.83) RD: 5.3% (–12.3–22.9%)	0.5046
	Yes	13 (20.0%)	11 (14.7%)		
Postoperative complications ^2^	No	58 (89.2%)	69 (92.0%)	RR: 1.35 (0.48–3.80) RD: 2.8% (–11.1–16.9%)	0.7900
	Yes	7 (10.8%)	6 (8.0%)		

Data are presented as *n* (%). Effect estimates are reported for the “Yes” category as risk ratio (RR) and risk difference (RD) with 95% confidence intervals (CI). *p* values were calculated using χ^2^ and adjusted for multiple comparisons using the Benjamini–Hochberg procedure. AABO, abdominal aorta balloon occlusion. ^1^ Intraoperative complications included bladder and intestinal injury. ^2^ Postoperative complications included arterial and venous thrombosis, acute kidney injury (AKI), hematometra, surgical site infection, intestinal paresis, and reintervention need.

## Data Availability

The datasets generated and analyzed during the current study are not publicly available due to institutional and ethical restrictions related to patient confidentiality, but they are available from the corresponding author upon reasonable request.

## Supplementary Materials

The following supporting information can be downloaded at: https://www.mdpi.com/article/10.3390/life16071106/s1, Supplementary Table S1. Adjusted analyses of intraoperative blood loss. Supplementary Table S2. Adjusted analyses of intraoperative total transfusion volume.

## Abbreviations

The following abbreviations are used in this manuscript:

PAS	Placenta accreta spectrum
AABO	Abdominal aortic balloon occlusion
RBCs	Red blood cells
RCT	Randomized controlled trial
ICU	Intensive care unit
