# Supplementary material for: Abdominal Aorta Balloon Occlusion Versus Standard Care in Placenta Accreta Spectrum Disorder: A Randomized Controlled Trial
Source: Life (Basel). 2026 Jul 2;16(7):1106. doi: 10.3390/life16071106 (PMC13413313; doi:10.3390/life16071106)
Supplement: Supplementary file 1 [file life-16-01106-s001.zip › life-4347510-supplementary.pdf]

Supplementary Table S1. Adjusted analyses of intraoperative blood loss

Model	Ratio of geometric means (95% CI)	β (log-scale)	Robust SE	p-value
Unadjusted AABO	0.66 (0.52, 0.83)	–0,420 (–0.649, –0.191)	0,117	0.0003
+ PAS grade	0.67 (0.55, 0.81)	–0,401 (–0.589, –0.212)	0,096	< 0.0001
+ PAS grade + prior CS	0.64 (0.53, 0.77)	–0,449 (–0.637, –0.261)	0,096	< 0.0001

Results derived from log-transformed linear regression with HC3 robust standard errors. Values <1 indicate lower intraoperative blood loss in the AABO group. Variance inflation factor for PAS grade and prior CS was 1.8, indicating no problematic multicollinearity.

Supplementary Table S2. Adjusted analyses of intraoperative total transfusion volume

Model	Ratio of geometric means (95% CI)	β (log-scale)	Robust SE	p-value
Unadjusted AABO	0.19 (0.06, 0.60)	–1,654 (–2.796, –0.512)	0,583	0.0045
+ PAS grade	0.21 (0.08, 0.55)	–1,566 (–2.531, –0.600)	0,493	0.0015
+ PAS grade + prior CS	0.16 (0.06, 0.43)	–1,813 (–2.790, –0.835)	0,499	0.0003

Results derived from log-transformed linear regression with HC3 robust standard errors. Values <1 indicate lower intraoperative transfusion requirements in the AABO group. Due to zero inflation (AABO: 26/65 [40%] received 0 mL; Control: 19/75 [25%] received 0 mL), log transformation used log(volume + 1). Results should be interpreted cautiously; the primary analysis of transfusion is presented in Table 3 (median difference in raw units).
